# Complete Genome Sequences of Microbacterium paraoxydans Phages Cassita and Fransoyer

**DOI:** 10.1128/mra.00885-22

**Published:** 2022-10-26

**Authors:** Brynn R. Bauer, Madelyn G. Brookins, Spencer Fobbe, Jade R. Fredrickson, Aidan D. Fretland, Nicole D. Grant, Abigail S. Katzenberger, Ali I. Khan, Brea L. Kieffer, Andrew M. Loken, Ignacio Lopez, Lindsay J. Lutton, Samantha A. Marquette, MaKayla J. Mears, Cadence M. Moe, Alexandra K. Parent, Rodrick P. Payne, Ida K. Peterson, Hailey L. Pucillo, Brina E. L. Rickman, Maddie A. Stubson, Elizabeth M. Zimmerman, Ashlyn M. Spring, Karen K. Klyczek

**Affiliations:** a Department of Biology, University of Wisconsin-River Falls, River Falls, Wisconsin, USA; DOE Joint Genome Institute

## Abstract

Phages Cassita and Fransoyer were isolated from soil in northwestern Wisconsin using Microbacterium paraoxydans as the host. The genomes of Cassita and Fransoyer are 61,868 bp and 62,277 bp, respectively, with direct terminal repeats. Both phages exhibit siphoviral morphology and are predicted to have lytic life cycles.

## ANNOUNCEMENT

**FIG 1:**
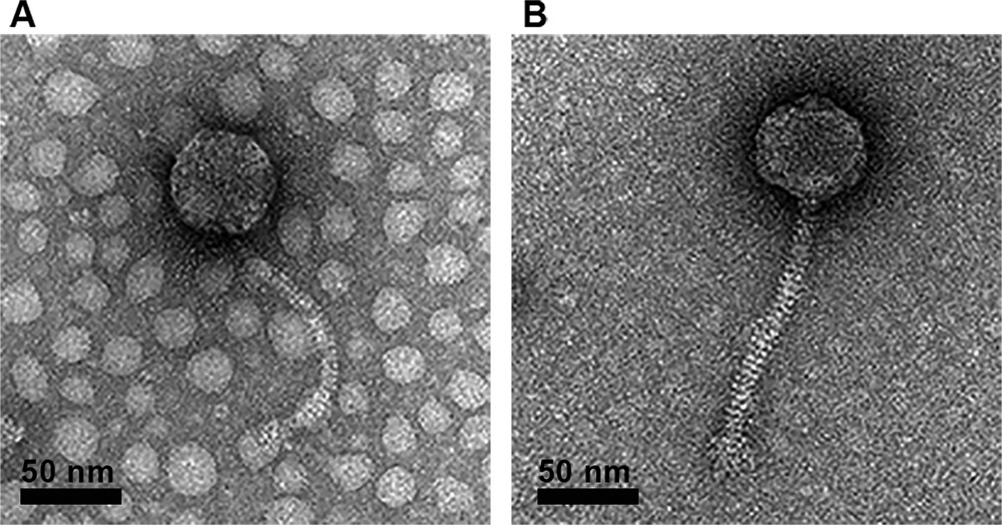
Transmission electron micrographs of Cassita (A) and Fransoyer (B). High-titer lysates were placed on Formvar-coated grids, negatively stained with 1% uranyl acetate (3), and imaged using a FEI Tecnai Spirit BioTwin transmission electron microscope at 120 kV. Cassita has a head diameter of 62 nm and a tail length of 167 nm (*n* = 1). Fransoyer has a capsid diameter of 60 to 62 nm and a tail length of 150 to 154 nm (*n* = 4).

Bacteriophages are the most abundant biological entities and represent a large reservoir of undiscovered genetic information (1). Analyzing the genomes of phages infecting a single host genus, such as *Microbacterium*, can provide insights into viral evolution and genetic diversity (2). Here, we report the genome sequences of two phages isolated on Microbacterium paraoxydans strain NRRL B-14843. Phages Cassita and Fransoyer were isolated from soil in northwestern Wisconsin (Table 1) using standard procedures (3). Briefly, soil samples were washed with peptone-yeast extract-calcium (PYCa) medium, the wash was collected by centrifugation and filtration (0.22-μm pore size), and the filtrate was inoculated with Microbacterium paraoxydans. Following incubation with shaking for 2 days at 30°C, the culture was filtered, and the filtrate was plated in PYCa top agar with Microbacterium paraoxydans, with three rounds of plaque purification. Both phages produced clear plaques of 1- to 2-mm diameter after 24 h at 30°C. Negative-staining transmission electron microscopy revealed that both phages have *Siphoviridae* morphology, with isometric capsids and long, flexible tails (Fig. 1).

**TABLE 1:** Genome characteristics of phages Cassita and Fransoyer

Phage name	Genome length (bp)	DTR^*a*^ length (bp)	G+C content (%)	No. of CDSs^*b*^	No. of tRNAs	Sampling location	Sampling location coordinates	Date of sample collection (mo/day/yr)
Cassita	61,868	1,649	57.9	130	1 (Leu)	Hudson, WI	44.9845N, 92.7545W	9/8/2021
Fransoyer	62,277	209	68.9	101	0	River Falls, WI	44.8419N, 92.6217W	9/13/2021

a DTR, direct terminal repeat.

b CDSs, coding DNA sequences.

Double-stranded DNA was isolated from phage lysates using the Promega Wizard DNA cleanup system, and sequencing libraries were prepared using the NEBNext Ultra II DNA library preparation kit. Sequencing was performed using an Illumina MiSeq system (v3 reagents), yielding 504,360 and 377,911 single-end 150-bp reads for Cassita (1,220-fold genome coverage) and Fransoyer (906-fold coverage), respectively. Raw reads were assembled using Newbler v2.9, and completeness was verified using Consed v29.0 (4). Sequencing results are reported in Table 1. Both genomes have defined ends with direct terminal repeats.

The genomes were annotated using DNA Master (http://cobamide2.bio.pitt.edu), PECAAN (https://blog.kbrinsgd.org), Glimmer v3.02 (5), GeneMark v2.5 (6), Starterator v1.1 (http://phages.wustl.edu/starterator), and Phamerator (7). Predicted gene functions were determined using BLASTp v2.9 (8), HHpred (9), TMHMM2 (https://services.healthtech.dtu.dk/service.php?TMHMM-2.0), and SOSUI (10), and tRNAs were identified using ARAGORN v1.2.38 (11) and tRNAscan-SE v3.0 (12). Default settings were used for all programs. Annotation revealed 130 protein-coding genes and one tRNA gene in the Cassita genome and 101 protein-coding genes in the Fransoyer genome. Both phages are predicted to have lytic life cycles, due to the absence of genes associated with lysogeny.

Cassita was assigned to cluster GB and Fransoyer to cluster EG, based on gene content similarity (GCS) of ≥35% to phages in the Actinobacteriophage Database (13, 14). Cassita shares 63.5 to 68.5% GCS with the three other phages in cluster GB, all of which were isolated on M. paraoxydans (14). We were able to assign putative functions for 35 of Cassita’s 130 genes. Twenty-four genes are unique, with no homologues in the database. Fransoyer shares >85% GCS with the cluster EG phages isolated on M. paraoxydans but <65% GCS with cluster EG phages isolated on Microbacterium foliorum (14). Some of the differences from M. foliorum phages are in genes predicted to encode minor tail proteins, which may play a role in currently unexplored host ranges (15). Fransoyer has four minor tail protein genes (genes 39 to 42), compared to three in the M. foliorum phage OneinaGillian (GenBank accession number MH727556) (genes 36 to 38) (14). Fransoyer gene 39 also has a 993-bp insertion relative to the homologous gene, OneinaGillian gene 36.

### Data availability.

For Cassita, the GenBank accession number is ON526969 and the Sequence Read Archive (SRA) accession number is SRX14443489. For Fransoyer, the GenBank accession number is ON645340 and the SRA accession number is SRX14443505.
